# Scapular Bronchogenic Cyst in a Girl Presenting as Recurrent Cellulitis: A Case Report and Review of the Literature

**DOI:** 10.1155/2018/7463724

**Published:** 2018-08-15

**Authors:** Zuhaib M. Mir, Ami Wang, Andrea Winthrop, Mila Kolar

**Affiliations:** ^1^Department of Surgery, Division of General Surgery, Queen's University and Kingston Health Sciences Centre, 76 Stuart Street, Kingston, ON, Canada; ^2^Department of Pathology and Molecular Medicine, Queen's University and Kingston Health Sciences Centre, 76 Stuart Street, Kingston, ON, Canada

## Abstract

Bronchogenic cysts are rare, congenital cysts originating from respiratory epithelium and typically found within the chest. Cutaneous bronchogenic cysts are exceedingly uncommon, with only 19 reported cases in the scapular region and almost exclusively occurring in male patients. Herein, we present the case of a female patient with recurrent cellulitis secondary to a bronchogenic cyst, which was diagnosed after surgical excision. We also provide a review of the literature to consolidate the current understanding of cutaneous scapular bronchogenic cysts. To our knowledge, this is the first such case reported from Canada.

## 1. Introduction

During normal embryologic development, the foregut divides into ventral and dorsal components, which go on to form the trachea and esophagus, respectively [1]. As development progresses, cartilage formation by surrounding mesenchymal cells leads to the establishment of the tracheobronchial tree [1, 2]. It is during this period of foregut development, where aberrant budding and branching is thought to lead to the formation of bronchogenic cysts. This certainly explains why the vast majority of bronchogenic cysts are located in the chest, either within the lung parenchyma or in the mediastinum; and these cysts may or may not be contiguous with the tracheobronchial tree [1–3]. Additional reported locations for bronchogenic cysts include cervical, paravertebral, scapular, pericardial, retroperitoneal, omental, and even perianal areas [4–8]. For scapular and cutaneous bronchogenic cysts specifically, postulated theories include aberrant migration of mesenchymal cells to the skin, metaplasia, or heterotopic differentiation of skin cells in situ [9, 10]. Nonetheless, a clear underlying mechanism has not yet been elucidated.

## 2. Case Report

Our patient is a 5-year-old female who was referred for evaluation and management of recurrent episodes of cellulitis in the left scapular region. A small cystic lesion had first been noted at the age of 2 years. While this had initially been an asymptomatic, small lump that grew in size over time, it first became symptomatic when the patient was 4 years old. The lesion developed surrounding induration and erythema, as well as purulent drainage and tenderness. Over the next 1-year period, she went on to develop 3 such episodes of cellulitis. This was treated with a 10-day course of cephalexin by the patient's family physician, and it was due to these recurrent episodes of infection that we saw the patient in consultation.

**Figure 1:**
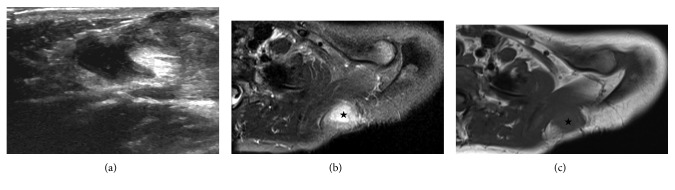
Radiologic images of the soft tissue lesion (star) from ultrasonography (a) and axial MRI ((b) *T1 weighted* and (c) *T1 fat suppressed*).

**Figure 2:**
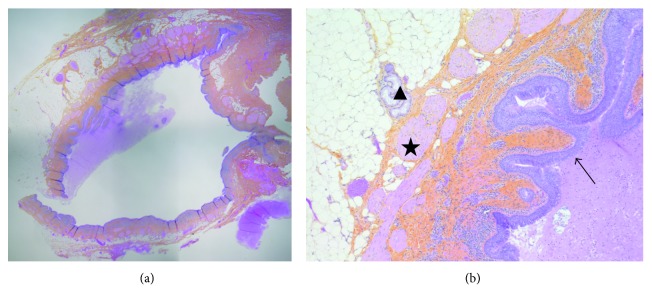
Representative sections of the bronchogenic cyst on routine H&E stain. (a) Low-power view of the subcutaneous cystic lesion (50x magnification) and (b) high-power view of the cyst wall, containing ectopic ciliated respiratory epithelium (arrow), smooth muscle (star), and small mucous glands (triangle) (200x magnification).

Physical examination revealed a playful and well-appearing 5-year-old female, weighing 24.8 kg and measuring 114.5 cm in height. Review of systems and cardiorespiratory examinations were unremarkable. On inspection of the left scapular region, approximately 7 × 5 cm area of cellulitis was noted, with a small opening and associated purulent drainage. The surrounding skin was tender to palpation, but there was no appreciable fluctuance. Thus, the initial working diagnosis was infected epidermoid cyst. An ultrasound of the affected area showed a complex cystic mass measuring 3.9 × 2.9 × 3.7 cm within the subcutaneous fat (Figure 1(a)). Deeper margins of the mass were poorly demarcated due to the degree of inflammation, and the lesion appeared to abut the underlying musculature. Since these findings were nonspecific, an MRI was obtained to further characterize the lesion. The MRI revealed the presence of a subcutaneous cystic lesion just superior to the scapula measuring 1.6 × 3.5 × 2.8 cm. While the mass abutted underlying muscular fascia, there was no extension into the underlying trapezius muscle itself (Figures 1(b) and 1(c)). Thus, the decision was made to pursue surgical excision of the lesion. This was performed under general anesthesia, which the patient tolerated well. Intraoperatively, we noted that the mass was quite soft, cystic, irregular in shape, and not well circumscribed. Nonetheless, we were able to excise the lesion in its entirety. Histopathology from this specimen revealed a benign cyst lined with ciliated columnar epithelium (Figure 2). In addition, the cyst wall itself contained smooth muscle, small mucous glands, and clusters of lymphocytes. Thus, these findings were in keeping with a bronchogenic cyst and not an epidermoid cyst as was initially suspected. On 1-year postoperative follow-up, the patient is doing well, with no recurrence of infection or other symptoms.

## 3. Discussion

Overall, bronchogenic cysts are quite rare and have a reported incidence of 1 in 42,000–68,000 [9, 11, 12]. Due to their infrequency and nonspecific presentation, we know that bronchogenic cysts are typically unrecognized and often diagnosed following surgical excision and histopathologic examination. They are characterized by a lining of respiratory tissue—ciliated cuboidal or columnar epithelium which may be pseudostratified—and they contain thick, mucoid material secreted from this lining [8, 10, 13]. Often, goblet cells, lymphoid aggregates, glands, and/or cartilage may also be found in association with the respiratory epithelium [8, 10, 13]. With respect to symptoms, some patients may present with an asymptomatic mass, or may complain of chest pain, cough, dyspnea, or fever [2, 3]. Clinical presentation as an abscess and/or infection has only been reported in a handful of cases in the literature so far [9, 14].

Scapular bronchogenic cysts are exceedingly uncommon, with 19 cases reported in the literature so far, and our case being the 20th [9–25]. The patient characteristics and findings from these cases are summarized in Table 1. The majority of scapular bronchogenic cysts occur in male patients (75%); however, there is no clear explanation for why this is. We also note that >50% of all scapular cysts occur on the left side. Again, there is no obvious reason for why this is the case. Predominantly, patients came to attention due to enlarging masses or draining sinuses in the scapular region. From our review of the literature, the natural history of scapular bronchogenic cysts involves enlargement of the mass with possibility for recurrent infection over time. As a result, these masses are often mistaken for sebaceous or epidermoid cysts and are treated as such. While there is minimal harm to this approach, there is at least 1 case report of a scapular bronchogenic cyst in a middle-aged man, which harboured a malignant melanoma, ultimately resulting in metastasis and death [16]. That being said, these cysts are generally not considered to be premalignant lesions. In addition, failure to recognize a bronchogenic cyst can delay definitive surgical excision, and subject patients to repeated incision and drainage procedures, as well as prolonged recurrent infections.

Our case is a unique presentation of a scapular bronchogenic cyst in a female patient as recurrent cellulitis. To our knowledge, this is the first such instance reported within Canada. Further research is required to elucidate the exact mechanisms underlying the formation of all bronchogenic cysts, and certainly, an index of suspicion should be reserved for this diagnosis when working up scapular lesions in children.

## Figures and Tables

**Table 1:** Reported cases of scapular bronchogenic cysts reported in the literature.

Case	Sex	Age at initial presentation	Initial presenting symptom	Side	Histopathology	Outcome	Reference
1	M	≤2 y	Mass	R	RE and smooth muscle	*Surgical excision and resolution*	Pul and Pul [17]
2	M	10 y	Mass	R	RE and lymphoid aggregates	*Surgical excision and resolution*	Das et al. [18]
3	M	≤2 y	Asymptomatic	L	Not documented	*Surgical excision and resolution*	Fraga et al. [10]
4	M	2 y	Asymptomatic	R	Not documented	*Surgical excision and resolution*	Fraga et al. [10]
5	M	≤2 y	Asymptomatic	L	Not documented	*Surgical excision and resolution*	Fraga et al. [10]
6	M	4 y	Mass	L	RE, goblet cells, smooth muscle, and mucous glands	*Surgical excision and resolution*	Yu et al. [19]
7	M	46 y	Growing mass	L	RE, sebaceous glands, squamous epithelium, and malignant melanoma	*Surgical excision, metastasis,* and *death*	Tanita et al. [16]
8	M	≤2 y	Growing mass	R	RE	*Surgical excision and resolution*	Tresser et al. [20]
9	M	≤2 y	Growing mass	L	RE, goblet cells, and smooth muscle	*Surgical excision and resolution*	Jona [13]
10	M	4 y	Mass	R	RE alternating with stratified squamous epithelium, goblet cells, sebaceous glands, and smooth muscle	*Incision and drainage, subsequent surgical excision*, *and resolution*	van der Putte and Toonstra [21]
11	F	8 y	Asymptomatic	^*∗*^	RE, goblet cells, and mucous glands	*Surgical excision* and *resolution*	Manconi et al. [22]
12	F	≤2 y	Draining sinus	L	RE alternating with stratified squamous epithelium, and mucous glands	*Surgical excision and resolution*	Ozel et al. [23]
13	M	3 y	Draining sinus	R	RE, stratified squamous epithelium, and lymphoid aggregates	*Surgical excision and resolution*	Kundal et al. [24]
14	M	3 y	Mass	L	RE	*Surgical excision and resolution*	Al-Balushi et al. [11]
15	F	5 y	Draining sinus	L	RE and lymphoid aggregates	*Surgical excision and resolution*	Farag et al. [12]
16	M	≤2 y	Abscess	R	RE, smooth muscle, squamous epithelium, and sebaceous glands	*Surgical excision and resolution*	Nakamura et al. [14]
17	F	4 y	Draining sinus	L	RE, smooth muscle, and seromucous glands	*Surgical excision and resolution*	Blanchard et al. [25]
18	M	3 y	Abscess	L	RE	*Surgical excision and resolution*	Zhu et al. [9]
19	M	≤2 y	Draining sinus	L	RE, squamous epithelium, goblet cells, smooth muscle, and seromucous glands	*Surgical excision and resolution*	Sun et al. [15]
20	F	5 y	Recurrent cellulitis	L	RE, smooth muscle, mucous glands, and lymphoid aggregates	*Surgical excision and resolution*	Current case

RE = respiratory epithelium (ciliated columnar or cuboidal epithelium +/− pseudostratified); L = left; R = right; ^*∗*^ = unknown, original article in Italian.
